# Intra- and inter-brain synchrony oscillations underlying social adjustment

**DOI:** 10.1038/s41598-023-38292-6

**Published:** 2023-07-11

**Authors:** Unai Vicente, Alberto Ara, Josep Marco-Pallarés

**Affiliations:** 1 Department of Cognition, Development and Educational Psychology, Faculty of Psychology, University of Barcelona, 08035 Barcelona, Spain; 2 Cognition and Brain Plasticity Unit, Bellvitge Biomedical Research Institute, 08907, L’Hospitalet de Llobregat, Spain; 3 Cognitive Neuroscience Unit, Montreal Neurological Institute, McGill University, H3A 2B4 Montreal, Canada; 4 BRAMS: International Laboratory for Brain, Music and Sound Research, H3C 3J7 Montreal, Canada

**Keywords:** Decision, Learning algorithms

## Abstract

Humans naturally synchronize their behavior with other people. However, although it happens almost automatically, adjusting behavior and conformity to others is a complex phenomenon whose neural mechanisms are still yet to be understood entirely. The present experiment aimed to study the oscillatory synchronization mechanisms underlying automatic dyadic convergence in an EEG hyperscanning experiment. Thirty-six people performed a cooperative decision-making task where dyads had to guess the correct position of a point on a line. A reinforcement learning algorithm was used to model different aspects of the participants’ behavior and their expectations of their peers. Intra- and inter-connectivity among electrode sites were assessed using inter-site phase clustering in three main frequency bands (theta, alpha, beta) using a two-level Bayesian mixed-effects modeling approach. The results showed two oscillatory synchronization dynamics related to attention and executive functions in alpha and reinforcement learning in theta. In addition, inter-brain synchrony was mainly driven by beta oscillations. This study contributes preliminary evidence on the phase-coherence mechanism underlying inter-personal behavioral adjustment.

## Introduction

Humans follow non-rational heuristics when making social decisions^1,2^. However, in contrast to other non-human primates (i.e., chimpanzes, bonobos), which maximize their own gains, humans are significantly more biased towards social leverage and fairness^3,4^. Social interactions require the coordination of distinct psychological functions resulting from diverse computations^5,6^ which occur in a reduced temporal scale^7^. Brain oscillations are the neural mechanism proposed to be suitable for information integration from different temporal scales and brain regions^8^. These oscillations have been identified as facilitators of dynamic temporal and spatial neural activity coordination mechanisms^8–11^ and apparently reflect distinct communication systems between areas in the cortex^12^. Particularly in the field of social neuroscience, neural dynamics and, more specifically, synchronic oscillations in interacting individuals, have been studied using two-person or multi-person approaches, in what has been named as “hyperscanning” settings. In these studies, it has been proposed that, in addition to the coordinated synchronization mechanisms of each individual's neural networks (intra-brain synchronization), oscillatory coupling between people (inter-brain synchronization) reflects the organization of collective behavior^13^. These mechanisms have been found, for example, in additive tasks (i.e., requiring joint effort; McGrath^14^), like joint-action or temporal motor synchronization^15,16^ but have also been observed in verbal interaction^17^, shared attention^18^ or intention^19^, creativity^20^ or decision-making^21^. In fact, despite ongoing debate on causality inferred by hyperscanning in inter-personal interaction^22–26^, there is evidence suggesting inter-brain phase synchronization is an index of collective performance^27^, sometimes providing an even better description of other self-report measures^28^. Synchronic coherence between two participants relates to other aspects of shared processing^29^. In particular, there is evidence of synchrony at alpha (8–12 Hz) and beta (13–25 Hz) in other paradigms such as in verbal interaction^17^ or alternating speech tasks^30^. Interestingly, in a recent massive sample (N = 4800) study conducted outside a lab (i.e., art installation in museums; Dikker et al.^31^), the authors reported inter-brain coupling in the beta band to be associated with joint social attention.

Despite its importance in understanding social behavior^6^, relatively few studies have investigated the adaptation resulting from socially induced adjustments such as conformity (see, e.g., Yu and Sun^32^). Conformity is a social adaptation that consists of adjusting one’s perspective and behavior to that of other people^33^ regardless of their rationale^34^. Furthermore, conformity is not only limited to norm deviation^33^ but may also be driven by the integration of different social sources of information^35^ that are related to individual or social contingencies (i.e., norms) or to potential gains in informational foraging^5,6,35^. According to this view, conformity may entail the convergence of responses to adapt to others’ decisions or views. This requires detecting discrepancies between one’s and another’s perspective, action selection, and mentalizing, among other social learning mechanisms^6^, as well as prediction and learning if this behavior is repeated^36^. Previous studies have shown the key role of oscillatory activity in these cognitive functions. For example, frontocentral theta oscillatory activity (4–8 Hz) has been proposed as a crucial neural mechanism of conflict and prediction error (PE) computation^37^–^39^. In this line, a study on MEG oscillatory dynamics of conformity has shown that theta oscillations track mismatched opinions between an individual and a group^40^.

Despite the importance of behavioral adjustments associated with social conformity, the oscillatory synchronization mechanisms involved in the processes necessary for aligning behavior with another person have yet to be discovered. To the best of our knowledge, no previous studies have investigated intra- and inter-brain synchronization in an experimental setting in which social conformity appears spontaneously. To fill this gap, we propose a novel experimental paradigm in which two people converge spontaneously in estimating a value without being instructed or extrinsically rewarded. To track expectations and prediction errors in the behavior of others’, we used a reinforcement learning (RL) algorithm that allowed assessing parameters on a trial-by-trial basis, as in previous research on social conformity (see e.g., Klucharev et al.^41^, Toelch and Dolan^5^, Bogdan et al.^42^). We hypothesized that theta oscillatory activity, which, as stated above, has been previously related to the computation of PE and cognitive control^37–39^ would be associated to synchronization of areas involved in cognitive control and behavioral adjustment. In addition, we hypothesized that alpha and beta bands, which have been consistently found when describing the neurophysiological correlates of social interaction behaviors, would be responsible for intra- and inter-brain synchronization.

## Results

### Trial-by-trial contrast analysis

**Figure 1:**
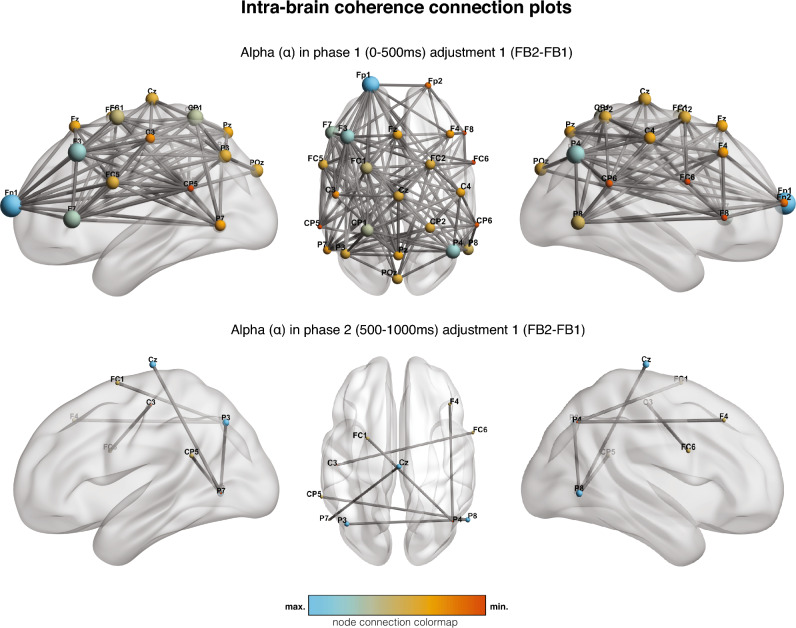
Intra-brain coherence connection maps in the alpha frequency range in the first (0–500 ms, top) and second (500–1000 ms, bottom) time ranges in the first adjustment (FB2–FB1). Color and size of each electrode sites indicate the number of credible connections with other electrodes, and the maximum and minimum are specific to each representation.

**Figure 2:**
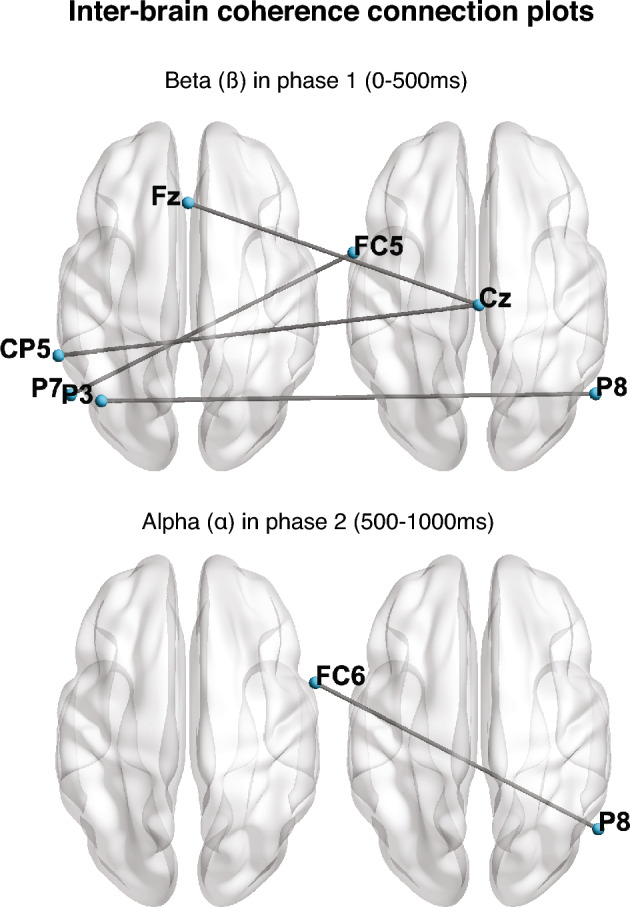
Inter-brain coherence connection maps in the beta frequency range (first time-range, 0–500 ms., top) and alpha (second time-range, 500–1000 ms., bottom) bands in the first adjustment (FB2-FB1).

Connectivity between electrodes was computed for each trial in three frequency bands (theta, alpha, and beta) and two time-ranges (0–500 ms. and 500–1000 ms. after stimuli). We report connections that present credible evidence of differences in coherent synchrony between conditions, both intra-personal and inter-personal. The intra-personal contrasts in the first feedback (FB) adjustment, the extraction of first feedback to second feedback (FB2-FB1), showed a credible change. In the first time range (0–500 ms.) we found credible connectivity associated with positive or increased activity in the alpha band (Fig. 1; 114/300 connections in HDI_NHCT_(95%) + ROPE). Inter-brain connections in the first adjustment were credibly and negatively related in beta (Fig. 2; 9/625 connections in HDI_NHCT_(95%) + ROPE). We found no sufficiently credible evidence of activity change regarding the second adjustment (FB3–FB2).

In the second time range (500–1000 ms.), the results also showed credible intra-personal frequency coherence related to a positive change in the first adjustment in alpha (4/300 connections in HDI_NHCT_(95%) + ROPE). In inter-brain data, we also found enhanced activity in alpha (2/625 connections in HDI_NHCT_(95%) + ROPE). Again, the second adjustment did not show a credible change in any of the studied frequency bands.

We also inspected adjustment (${FB}_{rep}\times Adjustment$), differentiating the lowest-adjustment trials (low-low contrast FB3–FB2) and the highest-adjustment trials (high-high contrast FB3–FB2). We only used the second adjustment contrast because FB2–FB1 was the reference in the identification of the adjustment level of the trial. The results did not reveal any credible or strong enough evidence related to the adjustment model (${FB}_{rep}\times Adjustment$) in any of the studied frequencies according to our proposed evidence criterion (HDI + ROPE rule).

### Reinforcement-learning analysis

**Figure 3:**
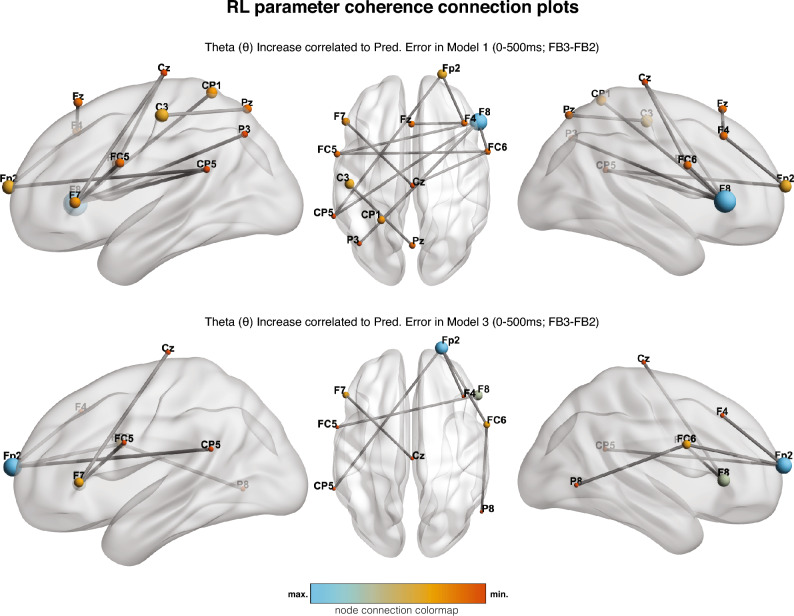
Intra-brain coherence connection maps showing an enhanced synchronization in theta band in the first phase (0–500 ms) of the second adjustment (FB3–FB2) for PEs (top) for Models 1 and 3. Color and size indicate the number of credible connections with other electrode sites, and the maximum and minimum are specific to each representation.

For each participant, a RL algorithm operationalized the willingness to cooperate with their peer, and the reward was modeled in three different ways (see Methods section), following the same update rule. We extracted the PE from these models to correlate it to phase connectivity, that is, changes at a single-trial level were contrasted with the connectivity measures. The results revealed a credible positive relationship with brain synchronization only in the first time range (0–500 ms.) for model 1 (M1; 14/300 connections in HDI_NHCT_(95%) + ROPE), which defines as a reward, the willingness to converge by the participant, and 3 (M3; Positive: 6 out of 300 connections in HDI_NHCT_(95%) + ROPE), which considers a reward as the degree of convergence continuously (Fig. 3). By contrast, we did not find any credible change in connectivity regarding the second model (M2), which considers reward as an adjustment, neither in the first nor in the second time range. Additionally, all the credible activity change after feedback related to PE (${FB}_{rep}\times PE$) was restricted to the theta frequency band in the second adjustment (FB3-FB2). Here, PE was associated with increased theta connectivity mainly in frontal areas according to M1 and M3, particularly in F8 and Fp2 electrode sites, plus a centroparietal cluster. Therefore, the results showed PE tracking in FB3-FB2, where responses can relate to previous adjustment learning. However, to test for differences between M1 and M3, we conducted a Tuckey pairwise analysis. We only found significant differences in the contrast between M1 and M3 with M2 (Table 1). BIC for the models is: BIC_M1_ = 275,38; BIC_M2_ = 285,26; BIC_M3_ = 274,98).

**Table 1:** Pairwise contrasts between Log-Likelihood scores of models 1 (M1), 2 (M2) and 3 (M3).

Contrast	Estimate	SE	Df	T-ratio	*p*-value
M1–M2	− 9.884	3.2	70	− 3.089	0.008
M1–M3	0.401	3.2	70	0.125	0.991
M2–M3	10.284	3.2	70	3.215	0.005

## Discussion

In the present study, we explored the intra- and inter-brain oscillatory phase-based connectivity in dyads during spontaneous behavioral adaptation in a social decision-making paradigm. At the intra-personal level, our results suggest local and distal neuronal population connectivity mechanisms in the alpha frequency band and a learning mechanism towards convergence tracked in the theta band. We also found inter-brain synchrony change in the dyads in the beta band. Lastly, we found credible differences in inter-personal oscillatory phase-based connectivity in alpha in the second time range.

Credible increased intra-personal brain frequency coherence changes in the first adjustment (FB2–FB1) were mainly and widely tracked in alpha in the first time-range (0–500 ms.), and a solid remanent was still tracked in alpha in the second time range (500–1000 ms.). We interpret this alpha synchronization change as caused by the broad inter-regional communication required for a multi-layered valuation, which could involve social (mirror neuron system, mentalizing) as well as non-social (attention, visual assessment, response planning, motor control, etc.) processes, or also both simultaneously. Differences in the phasic coherence were especially prominent in the first time-range (0–500 ms.), which supports its association with attention and cognitive control computations. Research shows that the alpha band plays a role in the top-down modulation of cognitive control functions (see Sadaghiani and Kleinschmidt^43^, for a review) and engagement in working memory^44^. In social neuroscience studies, alpha band involvement has also been reported to be associated with social cognitive processing^45,46^ and interactive decision-making^47,21^. The primarily cento-parietal alpha coupling activity remaining in the second-time range (500–1000 ms.) could also be associated with the proposed role of alpha in communicating task-relevant areas by inhibiting task-irrelevant ones^48^, which might help in the encoding, retention or recognition of information to transform sensory input into action preparation processes^49^. Significantly, while the first studied time range showed widespread alpha connectivity, the second time range was restricted to some fronto-centro-parietal electrodes. This could indicate either a progressive reduction of the role of the alpha oscillations as the main inter-regional communication mechanisms for longer latencies or a contribution in a more specialized function (e.g., selecting relevant information and inhibiting irrelevant ones, Park et al.^50^).

Recent evidence suggests alpha coherence is a large-scale rhythmic communication system between distant brain regions^51^. The results further support alpha’s role in orchestrating neural mechanisms underlying social adjustment.

As for inter-personal synchrony, the results show involvement of the beta frequency band, especially in frontocentral and centroparietal areas, in the first adjustment (FB2–FB1) and first time-range. Enhanced coupling of the beta band has consistently been found in social settings, such as action observation and imitation^52,53^ economic games^54^ and face-to-face interactions^31^. Given that some of these social settings involve joint actions, it has been proposed that this activity might be related to sensory-motor processes^55^ because beta activity is engaged in motor response and preparation^56^. However, even if a dyadic interaction and its regime of expectations require a particular sensorimotor engagement towards convergent action, this interpretation must be completed to explain situations that do not involve direct movement synchronization, as in the present study. Interestingly, a large-sample face-to-face hyperscanning study^31^ found that social traits like empathy and social closeness within partners predicted beta-band synchronization between dyads of people. The authors proposed that this activity could also be related to engagement and expectations about others’ actions. In the present study, participants generated expectations based on previous interactions with their peers, even if they could not stare at each other or talk. Despite such limited communication, we found inter-brain connectivity in the beta band at frontocentral and parietal sensors. This suggests that this activity goes beyond joint action and is relevant when evaluating others’ intentions and expectations. Aligned with this idea, Wang et al.^57^ found that frontal activity in the beta band during option evaluation predicted cooperative behavior in a computerized version of the Chicken Game^58^, where two players independently choose if they want to cooperate. Incidentally, Betti et al.^59^ presented evidence showing beta might have a role in integrating prior inferences with incoming information, which applies to the activity the mental activity of the participants at this moment. The authors suggested that the parietal attentional addition to a predictive comparison process in the frontal cortices might explain this frontal-parietal connectivity. Therefore, we suggest that our findings can be interpreted in light of this beta-driven socially related predictive computation, which does not necessarily require joint action or face-to-face interaction, even if it might enhance the engagement of these oscillatory mechanisms.

Another inter-brain coherent oscillation happens in the second interval with alpha involving connectivity between right frontocentral and right parietal electrodes. Dumas et al.^60^ were the first to report evidence of an inter-brain right centroparietal alpha oscillatory activity related to cooperation in face-to-face social communication. Interestingly, in our setting, such direct communication is blocked by a separator. Still, the inter-brain alpha coherence remains in a cooperation setting, suggesting that this activity could be associated with cooperative behaviors without requiring direct communication. This interpretation would be supported by the results of Szymansky et al.^27^, also showing an increase in alpha inter-brain coherence in cooperative conditions. However, although current results are coherent with previous literature, they should be interpreted cautiously as we only found an alpha inter-brain connection between two electrodes.

One prominent finding in the present study is the involvement of theta oscillations in computing PEs. We used an RL algorithm to model peer behavior predictions according to three distinct models. However, we only found credible evidence when defining reward as a cooperative change towards convergence, where PEs reflect the discrepancy from expected behavior (e.g., no convergence after trials of convergence; M1), and when defining reward as the closeness to convergence between participants (M3). Crucially, even when M1 shows broader frontocentral connectivity in the early time range (Fig. 3), the differences between both models are not significant according to their log-likelihood scores (Table 1). Notably, only theta coupling was engaged in this contrast. It was found in the first studied time range (but not in the latest), that is when usually the frontocentral theta oscillatory activity associated with performance monitoring and PE computation appears (e.g., Christie and Tata^61^; Cavanagh et al.^62^; Mas-Herrero & Marco-Pallarés^38^; van de Vijver et al.^39^). In addition, theta oscillations are involved in tracking complementary information in high-level RL computations^63,64^ and other low-level aspects like context uncertainty^62^,^38^. In addition, theta activity has been proposed as a critical brain mechanism in cognitive control^37^ regarding the comparison of expected with tangible outcomes and the synchronization of brain networks engaged for increased cognitive control. In the present study, the RL models allow us to assess the expectancies about the peers’ behavior affecting participants’ behavior. Therefore, if participants seek to converge, the estimation change largely depends on the previous convergence history between the participants. Deviations in peers’ behavior would require updating the current model and enhancing cognitive control mechanisms and coordination of brain areas through theta coupling^37^. Future studies with varying degrees of cognitive control allocation would contribute to understanding the functional role of these networks.

The present study is not exempt from limitations. First, although convergence was neither instructed nor directly induced, participants could feel that they should conform, likely due in part to the task structure (three repetitions of the same trial). A complementary explanation is that participants generally converge with their peers when they are embedded in social settings. It would be interesting to test this behavior when people are explicitly instructed to converge or are obliged to seek convergence. A second limitation is the high number of statistical tests performed in each condition and frequency (multiple-comparisons problem). We proposed a two-level statistical approach to study brain connectivity to tackle this issue. This allows using an accurate distribution of the data (beta distribution) besides attenuating the multiple-comparisons problem^65^. However, the hybrid use of a frequentist approach in the first level hampers its complete mitigation.

Furthermore, independent replications of the present results are necessary to support further or refute these results. In addition, we purposefully chose a dichotomous measure to guide our temporal difference in reward computation. Although the study aimed to investigate willingness to adjust to the peer to explain the behavioral adaptation of conformity, we acknowledge it is indeed a simplification of the participants’ regimes of expectations. Another possible factor that could influence the connectivity associated with the RL model could be the reaction times and/or the delays between the responses of the two participants. However, in the present experimental design, the feedback was not presented until the two participants wrote their estimations and pressed the intro button, making the interpretation of differences in reaction time not straightforward, and in addition, participants were not instructed about being fast or slow, only on being precise. Therefore, it would be interesting to explore the role of time in the connectivities found in the present paper with other experimental paradigms with greater control of the time that allow their inclusion as a random effect in the model. Furthermore, other individual differences have not been considered in the current manuscript and could be explored in future studies. Finally, the number of trials for a RL model is limited, as evidenced by the high BIC values. Notwithstanding caution in interpreting the results, these suggest enhanced theta phase synchronization related to PE in our task coherent with evidence as mentioned earlier^38,39,61,62^. Finally, we tried to be cautious in our interpretations of the inter-brain coherence results, as we acknowledge that the mechanical nature of this phase synchrony still needs to be clarified.

In conclusion, we present evidence of intra- and inter-personal phasic coherence in a conformity task at distinct brain regions, time ranges, and frequency bands. The results suggest specific roles for different oscillatory activities, with theta being primarily related to learning and cognitive control, alpha to higher-level control and communication between distal regions, and beta to inter-brain synchronization. This study contributes preliminary evidence on the phase-coherence mechanism underlying inter-personal behavioral adjustment.

## Methods

### Participants

36 participants in 18 dyads (random distribution of 20 women and 16 men; median age: 24 years old, range: 19–53 years old) took part in the study. All participants were adult volunteers and were rewarded with 30 euros for their participation. The experiment protocols were approved by the Institutional Review Board (IRB00003099) of the Bioethics Committee of the University of Barcelona. All methods were performed in accordance with the relevant guidelines and regulations. Informed consent was obtained from all participants.

### Procedure

**Figure 4:**
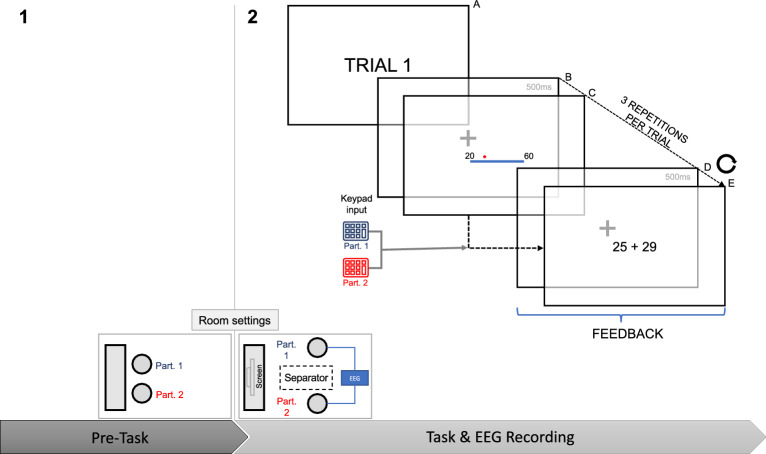
Diagram of experimental paradigm, which consists of two phases: the “pre-task” (first part, 1) and the main task with dual EEG recording (second part, 2). Below there is a representation of the room setting in these two phases. Participants (Part. 1 and Part. 2) shared the room in both phases, but a separator was added in the second phase to prevent communication. In the main task, every trial starts with an informative trial number (**A**), followed by a fixation cross for 500 ms (**B**), the stimulus participants used for their estimations (**C**) which is the moment when they had to use their keypads to introduce their responses, another fixation cross for 500 ms (**D**) and, finally, the feedback (**E**). Steps from B to E were repeated 3 consecutive times.

The experiment consisted of two parts (Fig. 4). First, each dyad completed cooperative tasks inspired by the cooperative dimension in the circumplex model^14^ at a maximum of one hour to pre-activate cooperative tendencies (i.e., intellective task, performance task, planning task, creativity task). After the activity, EEG was set up to record the dyads’ brain activity in the main task. As shown in Fig. 4, both participants shared the same room during the initial cooperative task and the subsequent main task phase. In the first part, they were able to interact. In the second part, a separator was introduced to block their ability to communicate or make eye contact with each other during the main EEG task.

In the main task, participants had to estimate the position of a point on a line. The line, either vertical or horizontal, and two numbers displayed at its two ends within the range of 0 and 150 and with a distance between them of 40–50 units, were simultaneously presented to both participants. Additionally, a red dot was positioned somewhere above the line, and the participants were instructed to indicate the dot's location using a numeric keypad and pressing the enter key after it. After the last participant pressed the enter key, the screen showed a fixation cross for a fixed 500 ms time pause. Then, the screen displayed the two participants’ estimations of the dot’s position until both participants pressed the return key (Feedback 1, FB1). Following this, participants repeated the same procedure two more times, observing the same line, numbers, and dot placement, providing their estimations, and viewing those of their peers (Feedback 2, FB2, and Feedback 3, FB3 for the second and third iterations). The moments of feedback (FB), wherein both participants had the opportunity to compare their estimations, were the key focus of the current study. It is important to note that participants were free to modify their estimates based on their peers' responses; however, this was not explicitly instructed or rewarded. The experiment consisted of four blocks of 25 trials each, with three repetitions per trial.

### Data collection

EEG was continuously recorded at a sampling rate of 1024 Hz using an ANT Neuro ASALab EEG amplifier from 25 scalp electrodes (Fp1/2, Fz, F3/4, F7/8, Fc1/2, Fc5/6, Cz, C3/4, Cp1/2, Cp5/6, Pz, P3/4, P7/8, POz, Oz) plus one on each mastoid (left and right) and two electrodes recording eye movements. A reference electrode was set on the tip of the nose. The electrode impedance was kept below 5kΩ throughout the experiment.

### Pre-processing

We used EEGLAB 2021.1 in MATLAB R2021a for pre-processing. The data was bandpass filtered from 1 to 42 Hz. Epochs from − 2 to 2 s were extracted from each trial, and independent component analysis (ICA) was applied to remove the eye and muscular artifacts. A Surface Laplacian spatial filter^66^ was applied to the data prior to angle extraction to mitigate the effect of volume conduction for electrode-level connectivity. We then subtracted the ERP from every single trial to ensure that frequency dynamics were task-related but not driven by the ERP. Subsequently, each trial was convolved using a complex Morlet wavelet. Angles of the wavelet coefficients were extracted for each single-trial time–frequency data point and used to compute synchronization between electrodes through inter-site phase clustering^66^ per frequency and trial. We then averaged over frequency bands (θ = 4–8 Hz; α = 8–13 Hz; β = 13–25 Hz) in two time ranges, from 0 to 500 ms. and 500–1000 ms., to study early and late processing, respectively. The rationale for dividing the analysis into these intervals was to study independently the early attentional and executive processes (which are reflected in feedback processing by the Feedback-Related Negativity/Reward Positivity and P3 ERPs, and by frontocentral theta oscillatory activity, which occurs approximately in the first 500 ms. after stimuli presentation, Ullsperger et al.^67^, Glazer et al.^68^, to the late mechanisms (indexed by, e.g., the late positive component starting at around 500 ms. after feedback presentation, Glazer et al.^68^).

### Two-level analysis

The statistical procedure followed a two-level analysis procedure^69^. In the first level analysis, we included ISPCs as dependent variables in a mass-univariate generalized linear model (beta-distributed values) and extracted their relation to the $FB$ repetitions (per dyad in the inter-personal analysis and per participant in the intra-personal analysis) using maximum likelihood estimation with the “glmmTMB” R package. Then, we used “lsmeans” R package to compute the least-squares means of the parameters of interest and extract their estimates and associated standard errors.

Subsequently, we used these first-level data to carry out null-hypothesis credibility testing (NHCT) in a second-level analysis. This was done by including the first-level data from all connections—excluding the mastoids (i.e., 625 connections in dyadic data, 300 connections in intra-personal data)—in a hierarchical Bayesian meta-analysis (BMA; Marsman et al.^70^; Kruschke and Lidell^71^) assuming normality (μ: identity; prior on σ: student-t, μ = 0, σ = 2.5, ν = 3) and with weakly informative priors over the intercept (normal, μ = 0, σ = 10 ), and over connections as varying effects (gamma, α = 1, β = 10) using the “brms” R package^72,73^). For NHCT, we consider posterior distributions credibly different than zero when the totality of a Region of Practical Equivalence (ROPE) around the null hypothesis (H0: β0 = 0), consisting of the range ± 0.01 ∗ SDy—where SDy is the standard deviation of the dependent variable, completely falls outside 95% of the posterior’s Highest Density Interval (HDI)^71,74,75^).

For the interaction analysis and the extraction of other contrasts of interest from the model (i.e., adjustments FB2–FB1 and FB3–FB2), we always took the estimates and standard deviations from the first level to the second. This means that contrasts and interaction terms were always calculated at the first level, so we only took the estimates and the standard deviations to the BMA for a sample-level analysis.

We assessed contrasts between the ISPC and feedback repetition (${FB}_{rep}$). We also examined the effect of adjustment type (Adj; ${FB}_{rep}\times Adj$), a dichotomization of $High$, (high-high, coming highly adjusted from the first opportunity to change their responses and continuing this way to the second) and $Low$ (low-low) adjustment, depending on the level of behavioral adjustment towards convergence effort in the trial compared to the median of all response adjustments by each participant in all trials. Connectivity representations were displayed using the BrainNet Viewer Tool^76^.

We analyzed differences between the first (FB1), second (FB2), and third feedbacks (FB3). It is worth noting that for differences between FB1 and FB2, we use FB2–FB1 contrast instead of FB1–FB2. The same applies to differences between FB2 and FB3. Bayesian hypothesis testing via parameter estimation has a fundamental advantage: we can compare the hypothesis to the region of a posteriori most probable values for the parameters the hypothesis targets. We sought to match the sign in the limits of HDI with the ongoing signal activity from one feedback to the other (i.e., a negative sign for a decrease in activity and a positive sign after an enhancement). Hence, we consider a relationship positive when the activity has increased in relation to its reference: e.g., a positive relationship in the contrast FB2-FB1 would indicate an increase in the synchronization of FB2 compared to FB1, whereas a negative relationship would be interpreted oppositely.

### Reinforcement learning analysis

In order to determine the expectancies of peers’ adjustment based on previous trials, we used a reinforcement learning (RL) algorithm^77^ to fit three different models, each considering a different reward ($r$) calculation to guide learning. At each time ($t$), the algorithm updated the subsequent $Q$ value using a simple Rescorla-Wagner^78^ learning rule where the value of the option ($k$) updates in response to $r$:

$${Q}_{t+1}^{k}={Q}_{t}^{k}+\alpha \times [{r}_{t}-{Q}_{t}^{k}]$$

In the model fit, the Q function was updated by the outcome (decision to cooperate or not by the peer, see below), but represented a state where the agent acts. With the intention of keeping the state representation simple, we used a dichotomous label per choice throughout the three models.

In the first model, we considered a choice as convergent ($r$ = 1) if the participant maintained their response and it was the same as their peers, or if the participant decided to change their response in pursuit of convergence with their partner for diverging responses; and a non-convergent ($r$ = -1) choice otherwise. This model focuses on the pursuit of convergence and rewards when agents’ action seeks convergence.

In the second model, we assessed the participants' level of adjustment or change, assuming that reward was associated with a greater degree of adjustment while penalizing uncooperative or less adjusting behavior. Therefore, if the analyzed participant had changed more than their partner, the behavior was rewarded ($r$ = 1); if the user changed less than their partner, the model penalized the behavior ($r$ = -1); if the two participants did not change, reward was set to 0 ($r$ = 0).

In the third and last model, the degree of convergence at every repetition defined the reward. Therefore, a decrease in convergence in the dyad was penalized ($r$ = − 1), and an increase in convergence was rewarded ($r$ = 1). In addition, if convergence in the following feedback was the same because the convergence was maximal (equal number in the two participants) reward was also maximum ($r$ = 1); and if the convergence was the same but not maximal, the reward was set to 0 ($r$ = 0).

For all values, the learning rate ($\alpha )$ and the temperature ($r$) were calculated via non-linear optimization (using *fmincon* function in MATLAB) per participant. τ controls the level of stochasticity, being τ = 0 completely random and τ = ∞ a totally deterministic choice. This parameter is used in the Softmax ($S$) choice rule, which converts values into action probabilities. Importantly, we decided to upper bound it to 2 (0 < τ ≤ 2) to maintain the monotonic relationship between $\alpha$ and τ as suggested in Zhang et al.^79^.

$${S}_{t}^{k}=\frac{{}^{\uptau {Q}_{t}^{k}}}{{\sum }_{i=1}^{k}{}^{\uptau {Q}_{t}^{i}}}$$

For the correlations with phase coherence, RL parameters were assessed in relation to the signal with the feedback repetition in interaction with the prediction error (${FB}_{rep}\times PE$). For model comparison, we used the Bayes Information Criterion ($BIC$), as suggested by Wilson and Collins^80^, using the following equation:

$$BIC=-2log\widehat{LL}+{k}_{m}log(T)$$

Here, $\widehat{LL}$ refers to the log-likelihood value at the best parameter fitting, ${k}_{m}$ is the number of parameters in the model ($m$) for the individual and $T$ is the number of trials.

## Data Availability

All data and the code to replicate the results reported in this manuscript are available at: https://doi.org/10.3886/E183301V3.
